# Robust alternative to the righting reflex to assess arousal in rodents

**DOI:** 10.1038/s41598-020-77162-3

**Published:** 2020-11-20

**Authors:** Sijia Gao, Diany Paola Calderon

**Affiliations:** 1 Department of Anesthesiology, Weill Cornell Medical College, New York, NY 10065 USA; 2 School of Electrical and Computer Engineering, Cornell University, New York, NY 10044 USA

**Keywords:** Diseases of the nervous system, Consciousness

## Abstract

The righting reflex (RR) is frequently used to assess level of arousal and applied to animal models of a range of neurological disorders. RR produces a binary result that, when positive, is used to infer restoration of consciousness, often without further behavioral corroboration. We find that RR is an unreliable metric for arousal/recovery of consciousness. Instead, cortical activity and motor behavior that accompany RR are a non-binary, superior criterion that accurately calibrates and establishes level of arousal in rodents.

## Introduction

**Figure 1:**
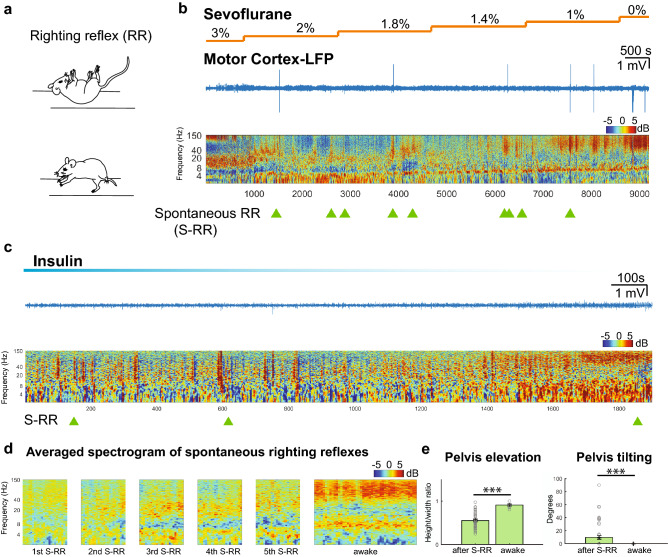
Spontaneous righting reflex is associated with low arousal state. (**a**) Schematic of spontaneous righting reflex. The animal is placed on its back and the subject rocks the trunk to the right and left side together with stretching of head and limbs. This results in rotation of the body so that all four limbs touch the ground (**b**) Representative trace of motor cortex raw LFP and normalized spectrogram during emergence from sevoflurane anesthesia. Color bar shows power in decibels. Light green triangles represent time points at which spontaneous righting reflexes (S-RR) were observed while the animal whose spectrogram is shown in the figure emerged from anesthesia and (**c**) hypoglycemic coma induced by injecting insulin (**d**) Average cortical spectrogram (60 s) at a time when the first five spontaneous righting reflexes occurred in animals exposed to anesthetic and insulin (n = 13 animals). Data was compared to the averaged spectrogram (120 s) obtained from the same group of animals once they regained full motor activity and wakefulness. (**e**) Quantification of pelvis elevation and tilting of the hips as an indirect measure of erect posture after S-RR. We measured the height/width ratio and the tilting angle of an ellipse that contours the animal hip and limbs (n = 52 S-RR; p = 0.0001 and *p* = 0.0001 respectively; Mann–Whitney U test and Two-Sample Proportion Test; See methods). ***p = 0.001.

RR is the gold standard for assessing arousal/recovery of consciousness (ROC) in rodents and relies on vestibular inputs that sense head movement, indicating awareness of surroundings^1,2^. The RR is assessed by placing the rodent on its back and measuring the time it takes for the animal to right itself (Fig. 1a). Inferences based on the RR test reflect the idea that wakefulness correlates with individual events^3–5^ including spontaneous movement of a body part^6^ or motor responses to painful stimuli^7^. When positive, RR represents restored consciousness^2^. Its simplicity means RR is widely used in studies of anesthesia reversal^3–5,8^, sedative EC50 compounds^9^, sepsis survival^10^, and post-hypoxia and traumatic brain injury^11^. Though used as a proxy for full recovery from unconsciousness, the righting reflex lacks cortical involvement^12^. It also persists after precollicular transections (absence of the telencephalon) in rodents^13^, and is retained in comatose-like rodents^14^. Such reports question whether a binary measure of ROC, such as the RR, truly distinguishes arousal/recovery of consciousness (ROC) from lack of consciousness in rodents.

## Results

RR-based metrics that are widely used to assess ROC in rodents, include its presence or absence per se^3,4,8,15–17^, the latency to first RR occurrence^2,5,18^, and tracking successive RRs as animals are repeatedly repositioned^16^. Using mice previously implanted with electrodes in the motor cortex area and carrying wireless EEG transmitters (Data Science International), we followed cortical activity and motor recovery during ROC in pharmacologically-induced coma models alongside conventional RR assays. When animals were exposed to a gradual reduction of sevoflurane (Fig. 1b) or during recovery from hypoglycemic coma (Fig. 1c), we observed multiple spontaneous-RR (S-RR) events. The first to the 5th S-RR occurred at cortical states with lower gamma power (40–150 Hz; average of n = 13 animals) than in awake, moving mice^7,19^ (1st-awake *p* = 0.001; 2nd-awake *p* = 0.001; 3rd-awake *p* = 0.015; 4th-awake 0.003; 5th-awake *p* = 0.01;Mann -Whitney U test) (Fig. 1d). Importantly, after S-RR, animals remained lying down (flattened posture; lowered pelvis) with insufficient hip swinging so that the hip was tilted relative to the surface (Fig. 1e. See methods). These mice were unable to hold up their body weight compared to awake mice, indicating that even when multiple S-RR events suggest ROC, animals have not reached expected levels of cortical arousal associated with this state. We further investigated this discrepancy.

**Figure 2:**
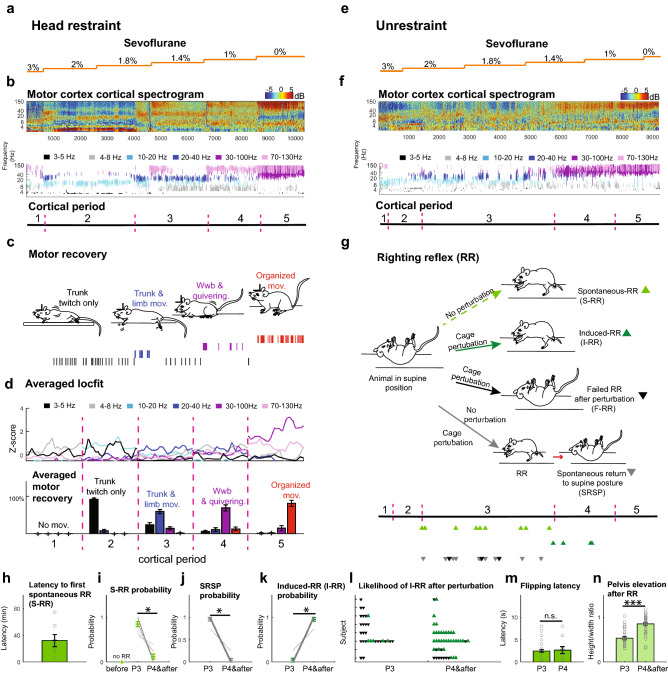
Cortico-motor features during emergence from anesthesia indicate RR is an ambiguous metric of arousal. (**a**) Sevoflurane concentration was ramped down (3% 1MAC to 0%) (**b**) Top: Normalized spectrogram (deviation from median) of raw LFP recorded in motor cortex (color bar shows power, dBs). Middle: changes in cortical state: black (3–5 Hz), gray (4–8 Hz), light blue (10–20 Hz), dark blue (20–40 Hz), purple (30–100 Hz) and lilac (70–130 Hz) after frequency clustering. Bottom: Cortical segmentation used a density estimation function and abrupt change detection algorithm (periods 1–5). (**c**) Movements during emergence from anesthesia include trunk-twitching (black lines), trunk/ hindlimb movements (blue lines), weak weight-bearing (Wwb) posture and quivering (purple lines), and organized movements (jumping, grooming etc., red lines). (**d**) Top: Averaged density estimation per cortical period (n = 13 animals; 500 s interval per period). Cortical periods(dominant frequencies; analysis outlined in methods). Bottom: Percentage of trunk twitching (91% ± 3.6%), trunk and limb movement (59% ± 5.4%), Weak weight-bearing (Wwb; 68% ± 7.8%), and organized movements (81% ± 7.9%). (**e**) Example raw LFP trace recorded in motor cortex during sevoflurane ramp down (orange line) in unrestrained mice. (**f**) Normalized spectrogram of cortical period clustering dominant frequencies (k-means /smoothed-Z score algorithm). Bottom: period segmentation obtained as in panel b (periods 1–5). (**g**) Top: RR events, including spontaneous RR (S-RR; light green triangle), induced RR (I-RR; dark green triangle), failed RR after perturbation (black triangle) and spontaneous return to a supine posture (SRSP; grey triangle). Bottom: Timepoints of RR event occurrence in example animal. (**h**) Latency from period 3 onset (P3) to first S-RR (n = 7 animals). (**i**) S-RR probability (n = 8 animals; *p* = 0.014; Paired Sample Wilcoxon Signed Ranks Test used in panels i-k). (**j**) probability of SRSP (n = 9 animals; *p* = 0.008) (**k**) I-RR probability during different cortical periods (n = 6 animals; p = 0.026). (**l**) Scatter plot showing the likelihood RR after perturbation. Triangle represent attempts to induce RR from P3 onward. (n = 8 animals). (**m**) Time from movement onset to supine to prone flip landing on four limbs (n = 39 vs n = 19; *p* = 0.88 *and p* = 0.03; Two-Sample Kolmogorov–Smirnov Test). (**n**) Quantification of pelvis elevation as a proxy for erect posture. (P3 n = 31 and P4&after n = 43; p =  < 0.001; Mann–Whitney U test). *p = 0.05, **p = 0.01, ***p = 0.001.

We next exposed animals to two inhaled anesthetics with different biophysical modulation^20^, sevoflurane (Fig. 2a) and isoflurane (Supplementary Fig. 2) and decreased anesthetic levels, gradually reducing the concentration to 0%. We analyzed cortical activity and motor behavior in detail, thus deploying measures used to follow recovery of a conscious state in humans^21,22^ to follow emergence from anesthesia in mice. Motor cortex activity was measured using bilateral electrodes to record local field potentials (LFPs) during anesthetic reduction (Fig. 2a) and up to 30 min after stopping treatment, allowing full expulsion of anesthetic^23^. We exposed C57BL/6 mice for 30 min to sevoflurane (n = 6) and isoflurane (n = 7) at a concentration of 3% and 1.25% vol. respectively (n = 13; males & females). In initial experiments, animals were head restrained (see methods) to minimize movement artifacts as animals emerged from anesthesia. In order to measure both the strength and frequency of motor movements, a two centimeter-piezo element sensitive to vibration was placed below the animal. This signal and cortical LFPs were simultaneously recorded (methods). Video and LFP recordings were synchronized to allow visual tracking of the animal. We applied a smoothing Z-score thresholding algorithm, which overcame variability evident in the cortical spectrogram in terms of the power of different frequencies (Fig. 2b). The algorithm allowed us to extract frequencies (Fig. 2b top panel, Fig. 2d top panel) occupying the most power (lower and upper limit frequencies were encompassed by a two-dimension vector; see methods). Possible cortical sub-states were then extracted through clustering of this two-dimension vector. This analysis revealed six clusters with six major frequency ranges (Fig. 2b middle panel,d top panel). Cluster robustness was tested by analyzing the sum of squared distances of samples to their closest centroid (inertia) (Supplementary Fig. 1).

3% sevoflurane (1 MAC, n = 6, Fig. 2b,d) immobilized the mice, and we observed 4–8 Hz (gray) was dominant, with high gamma (70-130 Hz;lilac) occurring intermittently. We defined this condition as period 1. Upon reducing sevoflurane, we observed increased power in the 3–5 Hz (black) and 10–20 Hz (light blue) bands, accompanied by brisk trunk twitching (distinct from breathing) and mild limb movements. We defined this condition as period 2 (Fig. 2b–d). Upon further anesthetic ramp down (1.8–1.4%), the 4–8 Hz (gray) and 10–20 Hz (light blue) bands persisted. Then bursts of the 20–40 Hz band (dark-blue) appeared accompanied by chirps in the 30–100 Hz (purple) and 70-130 Hz (lilac) bands. This cortical activity aligned with larger movements including trunk flexion, extension and limb abduction adduction (79.7%), and stronger and alternating limb movements (20.21%), (Fig. 2b–d; bottom panel). This combined cortical and motor activity characterizes period 3. The 30–100 Hz and the 70–130 Hz bands increasingly dominated together with the stronger presence of the 4–8 Hz band (Period 4; Fig. 2b–d). Accompanying this cortical pattern, the animals moved toward persistent partial support of body weight (squatting; Fig. 2c). Simultaneous movement of multiple body parts was seen, including quivering (a rapid full body twitch). However, when the 4–8 Hz power dominated in this period, the animals significantly reduced movement. Finally, gamma frequencies predominated (30-100 Hz & 70–130 Hz) and the alpha and beta bands significantly decreased as animals displayed an erect posture and organized, purposeful movements that progressed toward full weight-bearing posture, active jumping and grooming. This period persisted until we turned off the anesthetic and finished the recording. Note that detecting cortical features was not linked to the nominal value of the anesthetic concentration (Fig. 2a–d).

Cortical periods after exposing animals to isoflurane closely resembled those recorded in animals after treatment with and during recovery from sevoflurane (Supplementary Fig. 2b,c), with no significant differences in cortical period and movements (*F(9,160)* = 0.48, *p* = 0.8 Three-way ANOVA). Motor recovery showed a similar distribution across cortical features established following sevoflurane treatment (Supplementary Fig. 2b,c). While cortical responses differ slightly between anesthetics with similar MAC concentration (Fig. 2d, Supplementary Fig. 2c), these findings suggest that the integrated sequence of cortical and motor behaviors generalize across different anesthetics and are consistently observed as animals move from deep to light anesthesia (Supplementary Fig. 3).

**Figure 3:**
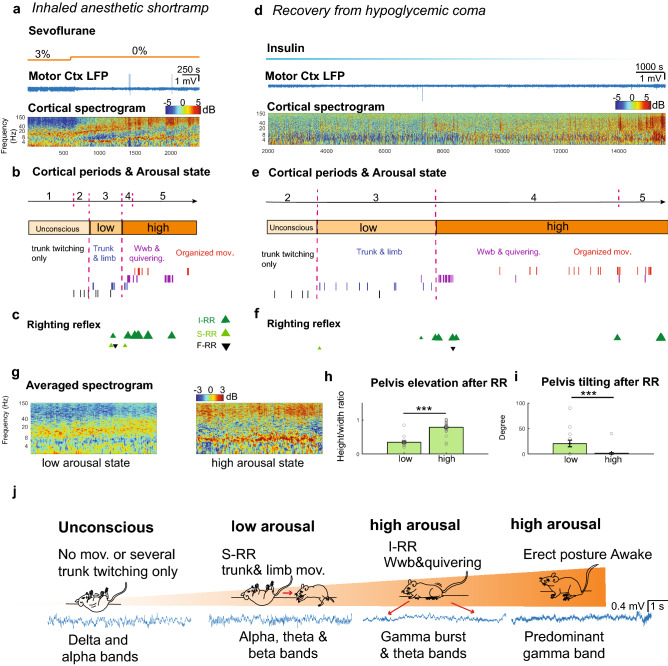
Integration of cortico-motor activity accurately determines the level of arousal. (**a**) Representative trace of LFP recorded in motor-cortex and normalized spectrogram during a short ramp of sevoflurane. Color bar represents power in decibels. (**b**) Segmented cortical periods and progression of motor behavior restoration defined high and low arousal states in the subject. (**c**) Distinct RR events including induced RR (I-RR), spontaneous RR (S-RR) and failed RR after perturbation (F-RR) during emergence from an animal exposed to a short ramp of anesthetic. (**d**) Motor cortex LFP and spectrogram of an animal recovering from hypoglycemic coma. (**e**) Segmented cortical periods and progression of motor behavior restoration define high and low arousal states in the hypoglycemic mouse (**f**) Dissimilar RR events observed during restoration of an awake state in an animal injected with insulin (**g**) Averaged spectrogram (1000 s) of motor cortical activity during a low (n = 5) and high (n = 5) arousal state. Color bar represents power in decibels. (**h**) Quantification of pelvis elevation and (**i**) tilting after RR in low (n = 17) and high arousal (n = 30); *p* <  = 0.001 and *p* = 0.0005; Mann–Whitney U test and Two-sample Proportion Test). ***p = 0.001. (**j**) Schematic illustrates levels of arousal defined by cortico-motor features during restoration of an awake state.

Having characterized cortical periods and motor recovery during emergence from anesthesia (Fig. 2a–d, Supplementary Fig. 2), we sought to establish the relationship between these features and the righting reflex.

Animals that had been implanted with cortical electrodes, were exposed to sevoflurane (Fig. 2e–g; n = 4) and isoflurane (n = 7; Supplementary Fig. 4), and then placed on their backs^13,24^ (Fig. 2g). A calibrated vaporizer in 50% air/50% O2 in a gas tight chamber was used to deliver a given anesthetic, and the same ramp that used as in head-restrained mice (above). We saw cortical and behavioral changes similar to those recorded in head-restrained mice (Fig. 2a–d). However, we also saw distinct head movements arising minutes after the initiation of trunk movements. Changes in both cortical signals and motor recovery tracked with those seen in restrained mice during periods 1–5 (Fig. 2b,f). No significant differences in terms of cortical period or movement progression was observed between restrained and unrestrained mice (*F(9,292)* = 1.35, *p* = 0.206;Three-way ANOVA) (Fig. 2d, Supplementary Fig. 4c).

After initiation of trunk (59.6 ± 8.3 min.) and limb movements (35.7 ± 10.5 min), animals began to spontaneously recover the righting reflex (Fig. 2g). The RR spontaneously arose 32.2 ± 9 min after the onset of the third period (Fig. 2h). RR had a higher probability of occurring during period 3 compared to other periods (Fig. 2i). Since the righting reflex is used to measure arousal, we expected RR to persist despite movement and external perturbation. However, the disorganized set of limb movements seen in period 3 led animals to spontaneously return to a supine posture (Fig. 2g,j). When we shook the chamber during period 3, animals showed a lower probability of flipping to a prone posture than in period 4 (Fig. 2k). Indeed, animals show multiple S-RR events in period 3 (Fig. 2g; 9 S-RR in a single animal). These results suggest that while the theta, alpha and beta band frequencies predominate, the righting reflex is transitory. This is puzzling as investigators often complete RR or score it as present (thus inferring recovery of consciousness) once they categorically observe the first spontaneous RR or several S-RRs^8,16,18^. Our data demonstrate that initial S-RR events occur at a low arousal state, and that S-RR does not predict recovery stage.

As we ramped down anesthetic and reached periods 4 and 5, RR loss was less likely (Fig. 2j). In addition, the probability of inducing a RR (I-RR) after shaking the chamber significantly increased compared to period 3 (Fig. 2k). Consistently, I-RR notoriously failed in period 3 when compared to period 4 (Fig. 2l). The increased likelihood of observing I-RR in period 4 suggests animals are more responsive to external stimuli. Interestingly, latency to flipping the body from supine to prone was indistinguishable between period 3 and 4(Fig. 2m). Lastly, the prone posture following the RR showed a significantly higher elevation of the pelvis relative to the surface in periods 4 and 5 compared to period 3 (Fig. 2n). In short, animals held their body weight on their limbs (erect posture) in period 4 compared to a flattened posture in period 3. Thus, although the RR itself is similar at different cortical levels, it is reversible when accompanied by a low-cortical activity state and animals laid down after the RR. However, once gamma frequencies begin to predominate (period 4), the RR is more likely to be induced and persists despite perturbation. Animals upstand after flipping. The fact that there are multiple RR events (Fig. 2g) and they are clearly distinguishable depending on cortical activity and motor behavior context demonstrates that RR alone is an imprecise measurement of arousal level. Therefore, RR is an unreliable metric of arousal and recovery of consciousness, especially when this response is assessed as a single event.

To demonstrate that integrating cortical activity and motor behavior accurately pinpoints arousal levels, we analyzed motor-cortical features obtained in long-lasting isoflurane and sevoflurane ramps under three conditions in which we often measure level of arousal and ROC. When applied to short ramps of anesthetic consisting of instantaneously switching concentrations from 3%-0% sevoflurane (Fig. 3a), cortical and behavioral events detected in long-lasting ramps were trackable despite rapid arousal (10 times faster than the prolonged anesthetic ramp; Fig. 3b). Similarly, we identified well-defined cortical periods and characteristic motor movements aligned with period 2–5 when animals recover from hypoglycemic coma (Fig. 3d,e) or from a bolus of propofol (Supplementary Fig. 5e). Combined spectrograms of short ramps (n = 3 animals) and hypoglycemic coma (n = 3 animals) during period 3 showed, on average, the predominance of theta, alpha and beta frequencies (Fig. 3g) together with flattened posture and tilted pelvis (Fig. 3h,i) indicating a low arousal state (Fig. 3b,e). In contrast, period 4 showed increased gamma power (Fig. 3g) together with quivering and erect posture (increased lifted pelvis; Fig. 3h) demonstrating a high arousal state (Fig. 3b,e). Cortical features in which dominant movements occurred were strikingly similar in propofol (Supplementary Fig. 5a–e). Using our proposed combined analysis, we were able to accurately calibrate arousal levels (Fig. 3b,e & Supplementary Fig. 5b,d) in multiple coma-like models, and use cortical and motor features to accurately assess ROC compared to RR (Fig. 3c,f & Supplementary Fig. 5c). Bigger triangles represent a higher pelvis elevation (erect posture).

## Discussion

Our experiments indicate that RR itself is indistinguishable across the different arousal states that occur during awakening. Moreover, we show that spontaneous return of righting reflex (S-RR) and consequential motor ability after animals flip to prone does not robustly predict recovery. However, our combined analysis capturing cortical and functional motor recovery can define the level of arousal at which distinct RR events occur. We note that no specific cortical signature is associated with the righting reflex. Since the righting reflex is a vague event during recovery from a comatose state, it is difficult to assess the onset of awakening using this metric. The fact that the cortical periods occur repeatedly across animals, treatments, and that they show the same sequential progression under different conditions (Supplementary Fig. 2) suggests that these periods follow a lawful progression of discrete states during the arousal process. The same applies to dominant motor behaviors (Fig. 2d, SFig 2c, SFig 4c, & SFig 5e).

Interestingly, the developing mouse embryo shows spontaneous movements which are very similar to those that occur during recovery from anesthesia and hypoglycemic coma. Prenatal movements transition from twitches of the trunk and limbs through to more complex movements that include lateral abduction as well as limb extension that progress toward coordinated movement (such as limb alternation)^25^. Such changes are observed in rodents and humans, and reflect caudal to rostral maturation that allow fetal responses to stimuli^26^. It is possible that arousal from the anesthetized or coma state in humans^21^ follows the same overall anatomical organization we are seeing in rodents.

Although researchers in the field recognize the significant limitations of the RR, the field has not provided a novel alternative to replace it. Indeed, current studies continue *to* actively and primarily use the RR. Even when RR is combined with cortical and behavioral changes, it remains a primary metric that researchers rely on during their first analysis step. As a result, investigators are only sampling a restricted range of recovered arousal. For instance, several studies show an increase in the theta/delta ratio^3–5,27^, reduced burst suppression ratio^28^, modest behavioral^29^ or cortical changes after stimulation^8^. However, they lack the presence of beta and gamma oscillations and purposeful movements characteristic of an awake state in humans^21^ or mice^30^ (Fig. 1d). A more recent study^31^ argued that since cortical gamma connectivity remains suppressed despite carbachol-induced wakefulness and that the changes in slow oscillations correlate with EEG activation rather than behavior, there is a need for reconsidering the role of EEG measures in monitoring consciousness. We provide a different perspective. Because the study arbitrarily defined full ROC as the restoration of the RR and uncoordinated limb mobility, we believe that such a behavioral operational approach cannot capture even a state that is verified as less than minimally conscious state^32^. Since recovery of the RR is relied upon, there is minimal activation in the beta and gamma frequency bands, and improvements are limited to an increase in theta/delta ratio and alpha rhythms. These results indicate that carbachol stimulation promotes partial cortical activation, leaving the exact state of the animals unclear. Application of our more fine-grained calibration of restoration would reduce confusion in the literature by carefully defining such states.

Our novel analysis defines cortical periods based on extracting the dominant frequencies (see methods*)* observed in the cortex as animals emerge from anesthesia or hypoglycemic coma. This smoothing procedure over the multi-stability allows us to equate our cortical periods to the metastable-states others have defined^6^. However, we acknowledge that additional dynamical details exist. Our approach offers context to the distinct phases of motor behavior. It demonstrates that animals emerging from diverse coma-like-states share a common dynamic process of cortical and motor arousal that is consistently sequenced from low to high arousal level (Supplementary Fig. 3). Thus, we provide a methodology to determine when integrative function has been regained in rodents.

We suggest that even when cortical activity measurement is impractical, behaviors such as weight-bearing on limbs, quivering, and standing posture after perturbation (erect vs. flattened) more precisely demarcate (Fig. 3j) the degree of awakening than the RR. It has been suggested that positional changes may have a significant impact on pathological behaviors^33^ and in vegetative and minimally conscious patients^34^. We observed a similar behavior in mice exposed to a pharmacological induced coma. Lifting the hip in mice indicates animals fully support their body weight on their limbs allowing the animal to execute locomotion. In our study, this posture strongly correlated with increased gamma frequencies (period 4 and 5) whereas lower frequencies such as alpha correlated with limb movements and a flattened posture.

Cortical activity and movement recovery reflect motor circuit restoration, including primary motor tracts, basal ganglia function, reticulospinal tract and spinal cord^21^ . Identifying cortical and motor features, as well as their interaction, will permit a greater understanding of how the brain recovers from different brain perturbations. Moreover, our combined analysis of motor-cortical activity offers a systematic calibration of cortical and behavioral features that truly identify the level of arousal and awakening in rodents as they recover from a deep coma state. This objective metric is both important for readers comparing studies in the area, and essential to determine if preclinical results can be genuinely translated from the bench to the bedside reducing potential inefficacious treatments, misdiagnosis in disorders of consciousness^32^, sepsis^10^ and brain injury^11^.

## Material and methods

### Stereotaxic surgery

All use of laboratory animals was consistent with the *Guide for the Care and Use of Laboratory Animals* and approved by the Weill Cornell IACUC (Protocol No. 2016-0055). Wildtype C57BL/6 mice at 10–18 weeks old were maintained on a reverse cycle, with food and water provided ad libitum. We used a total of 39 animals to perform the sevoflurane, isoflurane ramps, the propofol and the hypoglycemic coma experiments.

### Head holder implantation

Mice included both males and females and were anesthetized with isoflurane (n = 7) and sevoflurane (n = 6) in an induction chamber using an initial concentration of isoflurane (3%) or sevoflurane 5% respectively by volume in O_2_. Eyes were protected with ophthalmic ointment. Animals were then transferred to a stereotaxic frame, and anesthetic concentration was maintained using a nose cone. We monitored isoflurane or sevoflurane concentration using a gas analyzer (Riken Fi-I gas analyzer). The skull was fixed to the stereotaxic frame, the wound edge infiltrated with local anesthetic (bupivacaine 0.5%), and a designed-head holder device was placed on the skull to ensure durable head restraint without the need for ear bars (Gao et al.^30^). Two small craniotomies were made to target motor cortex (AP: 1.5–2 mm ML: ± 1.2 mm). In addition to the craniotomies, a stainless-steel reference screw was placed above the visual cortex. Craniotomies were covered with silicone for seven days and Flunixin 5 mg/Kg was administered subcutaneously.

### EEG transmitter implantation (EEG in unrestrained mice)

Animals exposed to isoflurane (n = 7) and sevoflurane (n = 7) were treated and maintained at a 1.25% and 3% concentration respectively (~ 1MAC). We applied local anesthetic as described before^35^. After opening an incision in the scalp, we created a subcutaneous pocket along the animal’s dorsal flank and placed the body of the transmitter (model F20-EET;DSI) into the pocket making sure biopotential leads were oriented cranially. Leads were placed in craniotomies made at stereotaxic coordinates targeting motor cortex bilaterally (AP: 1.5–2 mm ML: ± 1.2 mm). We secured leads using dental acrylic. Animals recovered for 7 days prior to experiments.

### Monitoring of head-restrained mice

Spontaneous ventilation was maintained throughout the experiment. Respiratory rate was continuously monitored by the investigators as described before^35^. Temperature was maintained at approximately 37 °C using a temperature regulator coupled to a rectal temperature probe (CWE Inc). We injected subcutaneous saline to maintain adequate hydration. After securing the animal’s head with the head holder, we removed the silicone applied to the craniotomies and implanted electrodes (tungsten electrodes, AM systems) to monitor cortical activity. Continuous field potentials in the cortex were recorded using the Plexon Omniplex System with Plexcontrol software (Plexon Inc., TX). To obtain the cortical field potential from wideband (0.2 Hz–40 kHz), we used a causal 4th order butterworth filter to minimize phase distortion as described before^30^. Signals were downsampled to 1 kHz. Using standard methodology, the terminally anesthetized animal was intracardially perfused with paraformaldehyde (4%), followed by brain extraction, post-fixation, microtome sectioning, and staining to confirm electrode placement.

### Monitoring of unrestrained mice

Animals were exposed to anesthetic delivered using a calibrated vaporizer in 50% air/50% O2 in a gas tight chamber. Temperature was maintained at approximately 37 °C using a temperature regulator (CWE Inc). We injected subcutaneous saline while the animal was deeply anesthetized to maintain adequate hydration. We monitored isoflurane or sevoflurane concentration within the chamber using a gas analyzer (Riken Fi-I gas analyzer). We placed chambers on to receivers and we used the Ponemah V5 software from DSI to record LFPs, counts of activity and temperature. Signals were down-sampled to 1 kHz.

### Righting reflex evaluation

We assessed the righting reflex (RR) by initially placing unrestrained mice on their backs (supine position). Then, we examined whether animals restore the RR (flip to prone position) and measured the time it takes for the animals to right themselves.

Spontaneous righting reflex (S-RR) was defined as a spontaneous return of the animal to a prone position per exposure. Note that we also considered S-RR a condition in which the animal spontaneously became supine again during the same exposure due to uncoordinated movement and minutes later, the animal spontaneously flipped again to a prone position.

Induced righting reflex (I-RR) was defined as a return of the animal to a prone position after regularly shaking the cage (approximately every 3 min) to place the animal in a supine position.

### Anesthetic ramps

Isoflurane anesthetic ramp: Animals were quickly induced with a concentration of isoflurane 3%. The anesthetic ramp was initiated upon exposing animals to isoflurane with a starting concentration of 1MAC (1.25%). Anesthetic concentration was reduced at intervals of 0.25% as previously described^35^. Each interval lasted for 30 min until reaching 0% anesthetic. For experiments using sevoflurane anesthetic ramps, animals were induced with a concentration of sevoflurane 5%. The anesthetic ramp was initiated upon exposing mice to sevoflurane 3%. Anesthetic concentration was reduced in intervals lasting half an hour each until the gas was turned off. Different animals were exposed to long anesthetic ramps. For short anesthetic ramps, 4 animals received repeated exposure of anesthetic seven days after finishing the long anesthetic ramp.

### Hypoglycemic coma

One week prior to inducing hypoglycemic coma animals were exposed to sevoflurane (n = 3) or isoflurane (n = 3), and the transmitter was implanted as described above. Hypoglycemic coma was induced using an intraperitoneal injection of insulin at 2–4 IU diluted in sterile saline (Humolin). Insulin concentration was titrated as described in Ref.^30^. Since we had implanted the DSI transmitter in advance, we could monitor the animals during hypoglycemic coma to check for and avoid abnormal epileptic activity. EEG activity was continuously monitored while animals lost the righting reflex and during restoration of an awake state. Figures show activity recording during recovery. All animals maintained spontaneous breathing.

### Propofol anesthesia

We implanted a jugular catheter and vascular access bottom in mice (n = 5) two weeks before the propofol injection. After anesthetizing mice with isoflurane, we first inserted the access bottom into the back of the animal. Then, we flipped the animal to a ventral position. We dissected the right jugular and inserted the catheter toward the heart. We used ligatures at the cranial and caudal end to secure the catheter and connected the bottom with the catheter. The catheter was weekly flushed with saline and locking solution (Heparin 500 IU/50% glycerol solution). A week before the propofol injection, we implanted transmitters as described above. The day of the experiment, we first flushed catheters and then applied a bolus of propofol (15 mg/kg/min; Fresenious Kabi) via a microinjector connected to a pump (World Precision Instruments) at a rate of 3.7 ml/min while we monitored brain activity. This concentration resulted in a deep anesthetic state in which animals breath spontaneously as described in^36^. We then assessed the emergence from anesthesia.

### LFP Spectral analysis

To examine changes in spectral content over time, we computed spectrograms using the Thomson multitaper method implemented in the Chronux toolbox^37,38^ in Matlab (Mathworks). We used the function *mtspecgramc* to compute cortical spectrogram with the following parameters: frequency band = 2–150 Hz, tapers = [3, 5], movingwin = [5, 2.5] seconds. For short ramp experiments, we used movingwin = [5,0.5] seconds. Spectral estimates were approximately chi-squared distributed, which is skewed^39,40^. Therefore we log-transformed (converted to dB) the fractional power and then removed its median over the whole trace^41^ . For insulin experiments, we used power and then subtracted its median as described^30^.

### Detection of dominant frequency band

We defined the dominant frequency band in a cortical spectrum as the band that surpasses other bands in power and that we previously described^35^. Briefly, it is identified by first measuring the mean power of each 50 log-spaced frequency^42^ between 2 and 150 Hz (denoted as $x=\left[{x}^{1},{x}^{2},\dots ,{x}^{50}\right]$) and then by detecting peaks in $x$ via a smoothed Z-score thresholding algorithm (stack overflow)^43^.

The smoothed Z-score thresholding algorithm takes $x$ as input and outputs a vector $y=\left[{y}^{1},{y}^{2},\dots ,{y}^{50}\right]$, which is a sequence of “0”, “1” or “− 1”. Zero represents no peak, -1 a negative peak or 1 a positive peak detected at a frequency span. In principle, peaks are identified by constructing a moving mean $\upmu$ and a moving standard deviation $\upsigma$ from a smoothed signal ${x}^{smooth}$. The algorithm requires 3 parameters to be specified: *lag* = represents how much of the data will be smoothed, specifically, number of last several datapoints in ${x}^{smooth}$ to update $\upmu$, $\upsigma$; *threshold* = deviation from $\upmu$ quantified in $\upsigma$ to notify a peak and *influence* (ranging between 0 and 1) = influence of new datapoints on ${x}^{smooth}$. In this paper, we set parameters *lag* = 5, *threshold* = 2 and *influence* = 0.1.

The algorithm is summarized as follows: We first initialized ${x}^{smooth}$ using the first *lag* number of datapoints in $x$ *and set* $\upmu$ = mean (${x}^{smooth}$), $\upsigma$ = std (${x}^{smooth}$).

For $j=lag+1 to N$ we did the following: If *abs* $\left({x}^{j}-\upmu \right)>$ *threshold* $\times\upsigma$, the algorithm signified a ‘1’ (positive) or ‘−1’ (negative). We then concatenated ${x}^{smooth}$ with a new datapoint = *influence* $\times {x}^{j}$ +(1-*influence*)$\times$ (last element in ${x}^{smooth}$). Likewise, if *abs* $\left({x}^{j}-\upmu \right)<$ *threshold* $\times\upsigma$, ${x}^{smooth}$ was concatenated by ${x}^{j}$. Finally, we updated $\upmu$, $\upsigma$ using the last *lag* number of datapoints in ${x}^{smooth}$. After obtaining $y=\left[{y}^{1},{y}^{2},\dots ,{y}^{50}\right]$, we first found ${y}^{i}$ in 3–5 Hz with maximum power > 4.5 dB. We zeroed ${y}^{i}$ with frequencies < 3 Hz to avoid artifacts often seen below this frequency, and then chose all spans of ‘1’s with length ≥ 5. The span with the highest mean power was determined as the single dominant frequency band.

### Classification of cortical states

We clustered span indices for dominant frequency bands in 3 animals during their emergence from isoflurane using K-means clustering and obtained 5 clusters: 4–8 Hz (theta), 10–20 Hz (alpha), 20–40 Hz (beta), 30–100 Hz (gamma), 70–130 Hz (high gamma). The optimal number of clusters is determined using the Elbow method on inertia (Fig. S1; wiki on elbow, inertia from python sklearn)^44^. To classify cortical states, we assigned the span index of dominant frequency bands to its nearest centroid.

### Segmentation of cortical period

We delineated cortical dynamics through occurrence density of the classified cortical states and then segmented this into periods by applying an abrupt change detection algorithm as we described earlier^35^. To achieve density estimation, we implemented the locfit.m function in the Chronux toolbox in MATLAB. In principle, time instants of a cortical state $i$ behave as single action potentials of a neuron. In the locfit.m function, we chose density estimation-type family = ``rate” and ``nearest neighbor” smoothing method with parameter 0.05. We interpolated locfit output using interp1.m function and applied z-score normalization to get the normalized density per cortical state per second. We segmented by first obtaining a matrix $A$ through concatenating normalized density of all cortical states (3–5 Hz, 4–8 Hz, 10–20 Hz, 20–40 Hz, 30–100 Hz and 70–130 Hz). Cortical states with number of time instants < 100 were discarded. We then implemented the findchangepts.m function (from Matlab) to find indices where local mean of $A$ changed most dramatically through minimizing the sum of residual error of each segmented region. We specified parameters ``MaxNumChanges” = 10 , ``MinDistance” = 600 for long ramp, hypoglycemic coma experiments and ``MaxNumChanges = 8″, ``MinDistance” = 150 for short ramp. Cortical periods were finally segmented based on these detected changepoints in a manual fashion.

### Computation of transition matrix for cortical periods

To determine whether brain activity is sequentially ordered in periods when emerging from anesthesia, we computed a transition matrix for cortical periods in isoflurane (n = 14 animals) and sevoflurane (n = 10 animals). We used Markov matrix modelling to describe fluctuations during cortical periods. This analysis establishes a relationship between the current state of a system with a previous state using a transition probability matrix. The element located at $i$th row, $j$th column in the transition matrix represents total number of transitions from period $i$ to $j$ observed on samples. We normalized rows in the transition matrix so that each row added up to one.

### Motor behavior observed from the video

Motor behavior was visually inspected by investigators blind to the experimental procedure. Motor behavior was classified as follows:

1. Trunk twitching.
2. Trunk and limb movement, including three type of movements and their combination:
  - fast erratic limb abduction, adduction, retraction and protraction of limbs.
  - limb alternation.
  - trunk extension, flexion and left and right rotation.
3. Quivering. Rapid full body twitching.
4. Organized Movement:
  - Full limb retraction with wide stand.
  - Active jumping.
  - grooming.

### Posture

Flattened posture (lowered pelvis). Animals lay down in prone position.

Weak weight bearing (elevated pelvis). This includes fast movement or dragging of the limbs in a wobbly squatting posture.

Fully erect posture (elevated pelvis). Abdomen is raised above the ground.

### Pelvis elevation and tilting estimation

To derive pelvic elevation and tilting after the RR, we took snapshots from videos using the software WondershareFilmora9. We drew an ellipse that contoured the animal’s hip and limbs in each of the snapshots using Illustrator. By applying properties to the ellipse, we quantified the ratio. Specifically, a height/width ratio higher than 0.8 signified pelvis elevation whereas a ratio less than 0.5 was classified as a flattened posture. Similarly, we estimated the tilting of the hip by determining the rotation of the longest axis of the ellipse with respect to an axis parallel to the ground plane.

### Movement detection using a vibration sensor

Animal movements were detected using a two centimeter-piezo element sensitive to vibration placed below the animal’s body as animals emerged from anesthesia and as we described before^30,35^. LFP signals and voltage changes as a result of vibration were simultaneously recorded using the Plexon OmniPlex system. We synchronized the LFP recordings with a video camera to observe the animal’s behavior.

### Statistical analyses for experiments

To study restoration of awakening, C-57/BL6 mice (male and females) were randomly assigned to isoflurane, sevoflurane, propofol and hypoglycemic coma groups.

For the statistical analysis (two-tailed) related to RR-events, we first applied the Lilliefors test to determine if the data was parametric. Since the data was non-parametric, we used the Paired Sample Wilcoxon Signed Ranks Test to examine disparate probability of RR-events across periods (Fig. 2i–k). In addition, we applied the Mann–Whitney U test to compare pelvis elevation (Fig. 1e, 2n, 3h) and Two-Sample Kolmogorov–Smirnov Test to compare flipping latency (Fig. 2m) in periods P3-P5. For pelvic tiling (Fig. 1e,3h), we implemented the Two-sample Proportion Test (with Fisher’s Exact Test method) to compare proportional samples with zero tilting angle in the posture following the righting reflex.

To examine whether there is consistency of motor behavior across periods between different anesthetics, we applied Three-Way ANOVA (Bonferroni Means Comparison) to investigate how factors “motor behavior” (4 levels), “period” (level = P2–5) and “anesthetic” (2 levels) affect movement percentage. We applied the same test between the restrained and unrestrained group.

### Sample sizes

To determine the sample sizes of the experimental groups we performed pilot experiments with 3 mice for the hypoglycemic experiments and for those mice exposed to prolonged and short anesthetic ramps. We considered the strength of the effect and the variance across the groups to determine the sample size (number of subjects). For experiments in which we assessed motor arousal, we estimated sample sizes using data previously published^6,30,45^. We estimated ideal samples by conducting power analysis. All experiments met or exceeded the ideal sample size.

## Supplementary information

Supplementary Figures.

## Data Availability

The data that support the findings of this study are available from the corresponding author upon reasonable request. The source data underlying Figs. 2b,f and 3a,d are provided as a source data file located at 10.6084/m9.figshare.12246296.

## Code availability

The function custom-written in Matlab to detect dominant frequency ranges during emergence from isoflurane in mice is available from the corresponding author.
