# Supplementary material for: Robust alternative to the righting reflex to assess arousal in rodents
Source: Sci Rep. 2020 Nov 20;10:20280. doi: 10.1038/s41598-020-77162-3 (PMC7679463; doi:10.1038/s41598-020-77162-3)
Supplement: Supplementary file 1 — Supplementary Figures. [file 41598_2020_77162_MOESM1_ESM.pdf]

Title: Robust alternative to the righting reflex to assess arousal in rodents

Authors: Sijia Gao[1,2] and Diany Paola Calderon[1*]

Affiliations:

1. Department of Anesthesiology, Weill Cornell Medical College, New York, New York, 10065.
2. School of Electrical and Computer Engineering, Cornell University, New York, New York, 10044.

Corresponding author:

(*) Please address correspondence to: Diany Paola Calderon MD. PhD.
Department of Anesthesiology
Weill Cornell Medical College
New York, NY, 10065
dpc2003@med.cornell.edu
tel. (212)746-4972
fax. (212)746-4879

Supplemental information

Figure legends

Supplementary Figures 1-5

Supplementary Figures

Supplementary Figure 1 Determination of optimal number of clusters for k-means clustering analysis. To determine the optimal number of clusters, we used the Elbow method to establish the number of clusters obtained from cortical activity of head-restrained animals emerging from (**a**) isoflurane and (**b**) sevoflurane. Inertia was computed as the sum of squared distances between samples and their closest centroids.

Supplementary Figure 2 Cortical periods and motor recovery prevail during emergence from isoflurane in head restrained mice. (a) Representative trace of LFP recorded in motor cortex while exposing a mouse to isoflurane anesthetic ramp. **(b)** Top: Normalized spectrogram of LFP. Medium: Cortical state after applying k-means clustering to principal frequency interval and using centroids obtained with isoflurane ramp animals. Bottom: Period segmentation and motor recovery. **(c)** Averaged density estimation per cortical state for each period (500 seconds interval) and the averaged percentage distribution of motor behavior while emerging from isoflurane (n=7 animals). Bottom: Averaged percentage distribution of trunk twitching (91%±5.4%), trunk and limb movement (66%±65%), weak weight-bearing (Wwb; 69%±11.6%), and organized movements (83%±12.6%).

Supplementary Figure.3 Cortical periods follow a consistent sequence when emerging from anesthesia. Transition matrix for cortical periods during the emergence

from anesthesia during prolonged anesthetic ramps of (**a**) isoflurane (n=9), (**b**) sevoflurane (n=6). Each row in transition matrix was normalized to reach one as a total.

Supplementary Figure.4 Resemblance of cortico-motor features in unrestrained animals emerging from isoflurane anesthesia. (a) Representative trace of LFP recorded in motor cortex while reducing isoflurane concentration. **(b)** Top: Normalized spectrogram of LFP. Medium: Progression of cortical states (clustered dominant frequency). Bottom: Segmentation of period and motor recovery. **(c)** Averaged density estimation per cortical state for each period (500 seconds interval) and the averaged percentage distribution of movement during anesthesia emergence in unrestrained mice (n=7 isoflurane ramp & n=4 sevoflurane ramp). Bottom:
Averaged percentage distribution of trunk twitching (87%±7.3%), limb movement (75%±3.7%), Wwb and quivering (55%±6.4%), and organized movements (65%±9.8%).

Supplementary Figure.5 Animals emerging from propofol progress through cortical and motor arousal features similar to inhaled anesthetics. (**a**) Representative trace of motor cortex LFP during propofol bolus injection (15mg/kg; purple line) & normalized spectrogram of LFP. (**b**) Segmented cortical periods and progression of motor behavior restoration defined high and low arousal states in the subject. (**c**) Distinct RR events including I-RR (light green) and S-RR (dark green) during emergence from propofol exposure. (**d**) Averaged spectrogram (1000 s) of motor cortical activity during a low (n=5) and high (n=5) arousal state. Color bar represents

power in decibels. (**e**) Quantification of pelvis elevation as an indirect measure of erect posture in low and high arousal state. ***p*=0.01; Mann–Whitney U test. (**f**) Averaged density estimation per cortical state for each period (500 seconds interval) and the averaged percentage distribution of motor behavior while emerging from propofol (n=5 animals).

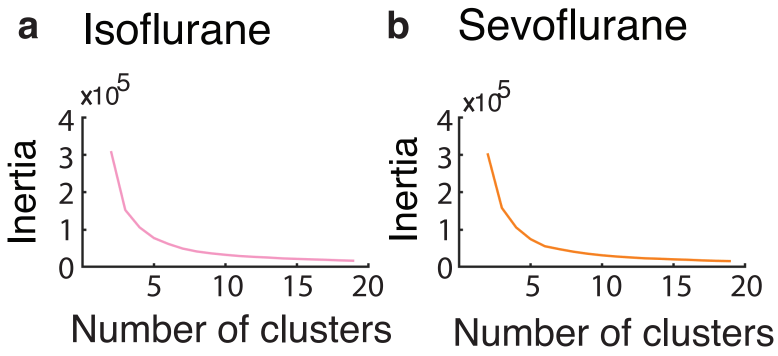

Supplementary Figure 1

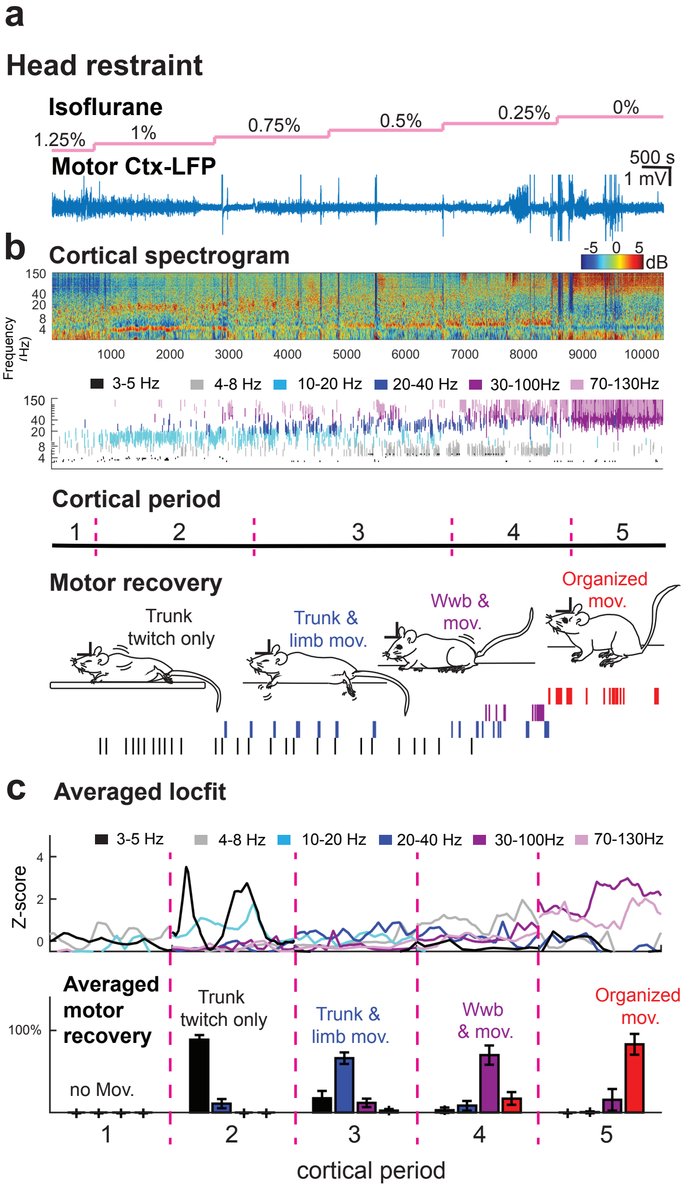

Supplementary Figure. 2

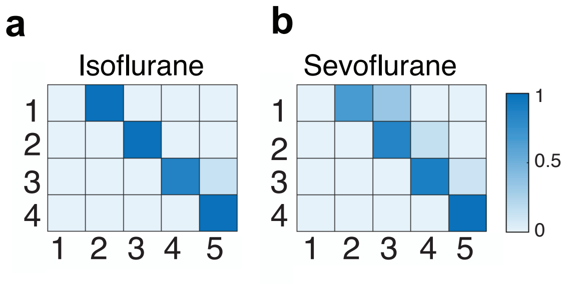

Supplementary Figure. 3

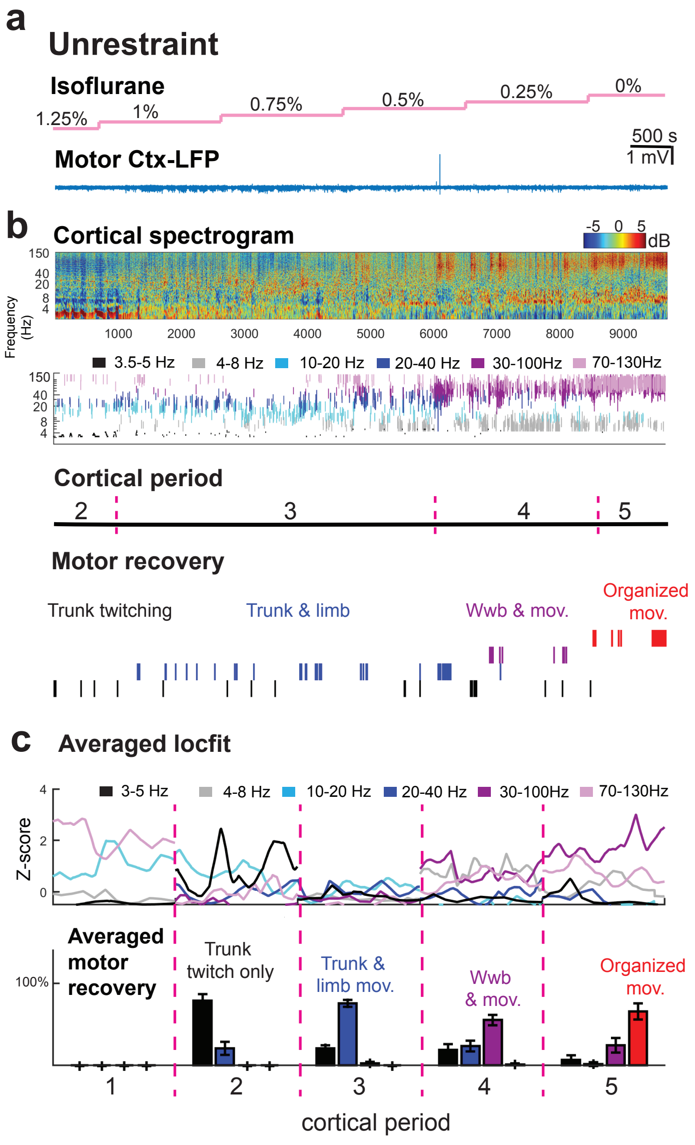

Supplementary Figure 4

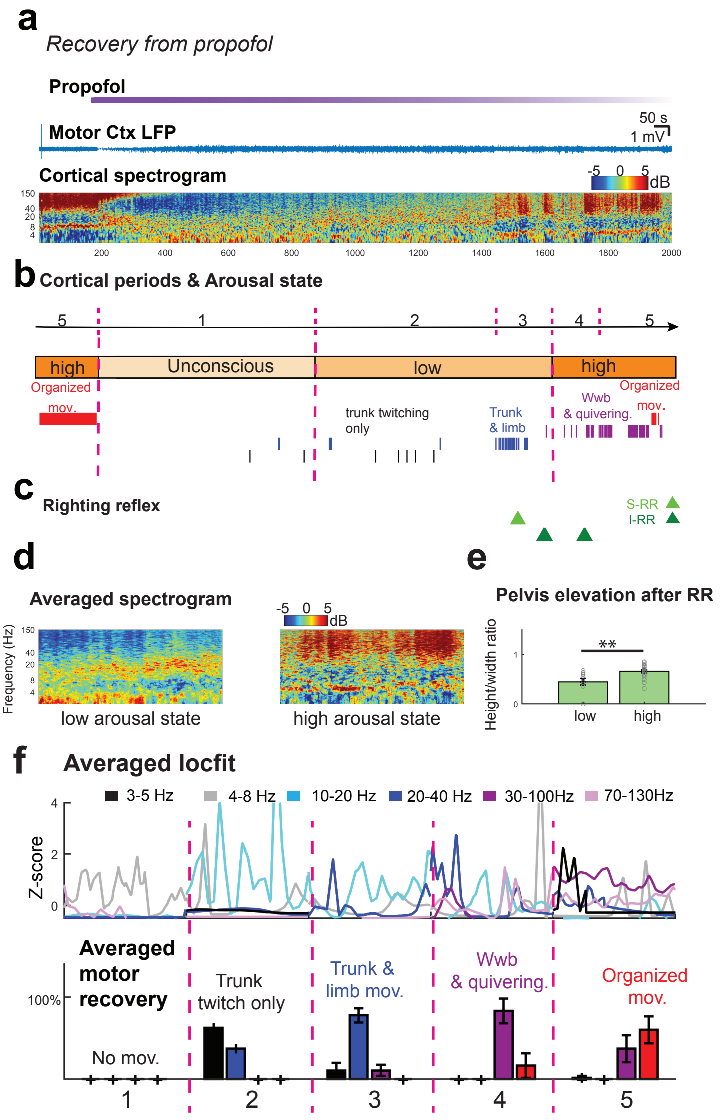

Supplementary Figure 5
